# Loop-Mediated Isothermal Amplification (LAMP) for the Diagnosis of High-Burden Viral Diseases in Resource-Limited Countries

**DOI:** 10.3390/pathogens15030248

**Published:** 2026-02-26

**Authors:** Ana Catharina Vasconcelos-Martins, Marta Giovanetti, Maria Carolina Elias, Svetoslav Nanev Slavov, Sandra Coccuzzo Sampaio

**Affiliations:** 1 Center for Viral Surveillance and Serological Assessment (CeVIVAS), Instituto Butantan, São Paulo 05503-900, Brazil; a.martins.proppg@proppg.butantan.gov.br (A.C.V.-M.); carolina.eliassabbaga@butantan.gov.br (M.C.E.); 2 Instituto Butantan, São Paulo 05503-900, Brazil; 3 Department of Sciences and Technologies for Sustainable Development and One Health, Università Campus Bio-Medico di Roma, 00128 Rome, Italy; giovanetti.marta@gmail.com; 4 Instituto René Rachou, Fundação Oswaldo Cruz, Belo Horizonte 30190-002, Brazil; 5 Climate Amplified Diseases and Epidemics (CLIMADE) Americas, Belo Horizonte 30190-002, Brazil

**Keywords:** Loop-mediated isothermal amplification, LAMP, nucleic acid amplification, dengue, influenza, Limited-resource settings

## Abstract

Loop-mediated isothermal amplification (LAMP) is an innovative nucleic acid amplification technique that operates under isothermal conditions and is distinguished by its high analytical efficiency, cost-effectiveness, and operational simplicity. Unlike conventional molecular assays, LAMP does not require sophisticated instrumentation or highly specialized personnel, rendering it particularly suitable for diagnostic deployment in resource-limited settings. Reaction outcomes are typically determined through direct visual inspection, often via colorimetric readouts, further enhancing its applicability in decentralized and point-of-care contexts. Owing to these attributes, LAMP has emerged as a valuable tool for the diagnosis of infectious diseases, particularly in regions with constrained laboratory infrastructure. Its affordability, rapid turnaround time, and ease of implementation support large-scale testing during public health emergencies, including epidemics and outbreaks, thereby contributing to the reduction in disease burden. Timely and accurate pathogen detection using LAMP can substantially strengthen public health responses aimed at controlling and mitigating viral transmission. This review provides an overview of the LAMP methodology, with an emphasis on its application in the detection of viral pathogens with epidemic and pandemic potential. Dengue virus and influenza virus are discussed as representative model infections to illustrate the diagnostic performance and practical advantages of LAMP-based assays. In addition, we explore current challenges and future perspectives for the implementation of LAMP in resource-limited settings, highlighting the need for continued technological refinement and contextual adaptation to maximize its impact on global health initiatives.

## 1. Introduction

Loop-mediated isothermal amplification (LAMP) is an isothermal nucleic acid amplification method developed by Notomi and collaborators, together with Eiken Chemical Co., at the University of Tokyo, and Osaka University Medical School [1]. Although initially established between 1998 and 2000, LAMP gained widespread recognition in 2003 during the emergence of the West Nile virus and Severe Acute Respiratory Syndrome Coronavirus (SARS-CoV), where it was applied for rapid diagnosis [2,3,4].

The key characteristics of LAMP include its rapid turnaround time (between 15 and 30 min) and high specificity and sensitivity, capable of detecting low copy numbers of nucleic acids [1,5]. Unlike conventional PCR variants, LAMP operates at a constant temperature, requiring primer sets and a polymerase, eliminating the need for thermal cycling equipment and highly trained laboratory personnel [5].

The high specificity of LAMP is attributed to the use of several primer sets that target different portions of the region of interest. By the time of its introduction, the method applied four primers: the forward inner primer (FIP), backward inner primer (BIP), forward outer primer (F3), and backward outer primer (B3). In 2002, Nagamine introduced two additional primers, the loop forward primer (LF) and loop backward primer (LB), to enhance amplification speed [6]. The polymerase, originally derived from *Geobacillus stearothermophilus* (BST polymerase), has also been improved, with variations like BST, BST-2.0, and BST-3.0, offering higher reaction efficiency and, at the same time, resistance to PCR inhibitors [7]. Positive amplification is typically detected visually through colorimetric changes in the reaction mix, facilitated by dyes that interact with reaction by-products or the amplified product itself [8].

Compared to other isothermal methods, LAMP occupies an intermediate position with respect to assay complexity, robustness, and analytical performance. Recombinase polymerase amplification (RPA) is a highly sensitive and selective isothermal technique that is faster than LAMP and operates at lower temperatures (37–42 °C). However, RPA presents several limitations, including the frequent need for post-amplification processing steps such as purification, inhibition at high DNA concentrations, extensive primer optimization due to the lack of dedicated primer design tools, and intrinsic difficulties in multiplexing [9]. Helicase-dependent amplification (HDA), while conceptually simple and closely mimicking natural DNA replication, exhibits low efficiency for the amplification of long amplicons, limited thermal stability and shelf life of the enzymes employed, and reduced assay sensitivity at lower operating temperatures. Owing to these constraints, HDA has shown slower adoption as a diagnostic platform when compared with LAMP-based assays [10]. In contrast, nucleic acid sequence-based amplification (NASBA) is restricted to RNA targets and, relative to LAMP, requires multiple enzymes—including T7 RNA polymerase, RNase H, and avian myeloblastosis virus reverse transcriptase—as well as stringent reaction control, substantially limiting its operational robustness [11]. Finally, more recent CRISPR-based diagnostic approaches enhance sequence specificity through programmable nuclease activity. Nevertheless, these methods typically involve lengthy sample processing workflows, frequently depend on specialized instrumentation, and require reagents that must be transported and stored under ultralow-temperature conditions. These factors collectively constrain the applicability of these assays as point-of-care diagnostics and their deployment in field settings [12]. In this isothermal landscape, LAMP represents a pragmatic balance between sensitivity, operational simplicity, and scalability, although important translational and implementation challenges may exist.

The simplicity and cost-effectiveness of LAMP make it particularly suitable for detecting a wide range of infectious agents in resource-limited settings, particularly in hospital environments [13].

Resource-limited settings can be regarded as diagnostic environments that represent limited access to reliable-electricity, cold-chain infrastructure, trained laboratory personnel, and formal quality assurance systems often combined with constraints in equipment availability and supply logistics. They encompass heterogeneous diagnostic environments, and the practical value of LAMP varies accordingly. In primary healthcare facilities, basic laboratory infrastructure and trained personnel may be available; however, access to high-cost reagents, appropriate cold-chain storage, and thermal cyclers is often limited. In such scenarios, LAMP provides a rapid alternative for viral detection with minimal equipment requirements. Its high analytical sensitivity and tolerance to moderate variations in reaction conditions support near-patient testing, although contamination control and the implementation of standardized workflows remain critical challenges [14,15].

In field-based surveillance contexts—such as outbreak investigations or plant and veterinary disease monitoring—portability, rapid turnaround time, and visual or instrument-free readouts are essential. Under these conditions, precise temperature control is often impractical, and LAMP assays, particularly when coupled with colorimetric or lateral-flow detection formats, are especially advantageous. These configurations enable large-scale screening without continuous power supply or complex instrumentation, as demonstrated by their application in plant disease surveillance and the detection of zoonotic viruses in animal populations [16,17].

As model pathogens for LAMP detection in this review, we chose dengue (DENV) and influenza viruses. These infectious agents pose complementary and clinically relevant challenges for isothermal RNA diagnostics. Both viruses require reverse transcription for detection, yet they differ substantially in genomic organization, evolutionary dynamics, and, critically, epidemiological context. DENV, a tropical vector-borne pathogen, comprises four distinct serotypes and multiple genotypes, which complicates primer design, particularly when simultaneous detection of genetically diverse strains within a single reaction is required. Dengue fever exerts a disproportionate burden in low- and middle-income countries, where it can drive explosive outbreaks associated with high morbidity and mortality. In such settings, affordable and sensitive diagnostic tools are essential to inform timely public health responses and outbreak control strategies [18,19,20]. In contrast, influenza viruses possess a segmented, negative-sense RNA genome and evolve rapidly through antigenic drift and genomic reassortment, frequently resulting in the emergence of novel strains and the replacement of circulating lineages. Influenza causes recurrent global epidemics and pandemics, exhibits marked seasonality, and represents one of the leading causes of respiratory disease burden worldwide, including in resource-limited settings. As with DENV, accurate and accessible diagnostics are central to clinical management and public health decision-making [21,22,23]. Collectively, these characteristics enable a rigorous assessment of LAMP performance across genetically diverse and rapidly evolving RNA viruses of major global public health relevance, thereby extending the significance of this review beyond any single viral system.

## 2. Brief Overview of the Reaction Mechanism

LAMP reaction requires two to three primer sets with two primer sets strictly necessary for amplification. The primers target distinct genetic regions, making the technique highly specific [24]. The two inner primers, FIP and BIP, possess a hybrid structure and are composed of two distinct portions. The FIP consists of the F2 sequence, which is complementary to the target DNA, and the F1c sequence, which is identical to the target region required for loop formation. Similarly, the BIP comprises the B2 and B1c regions. The outer primers, F3 and B3, anneal to regions located outside the binding sites of the inner primers. In addition, two optional loop primers—LF and LB—may be included to accelerate the amplification reaction by hybridizing to the formed stem–loop structures [25].

**Figure 1:**
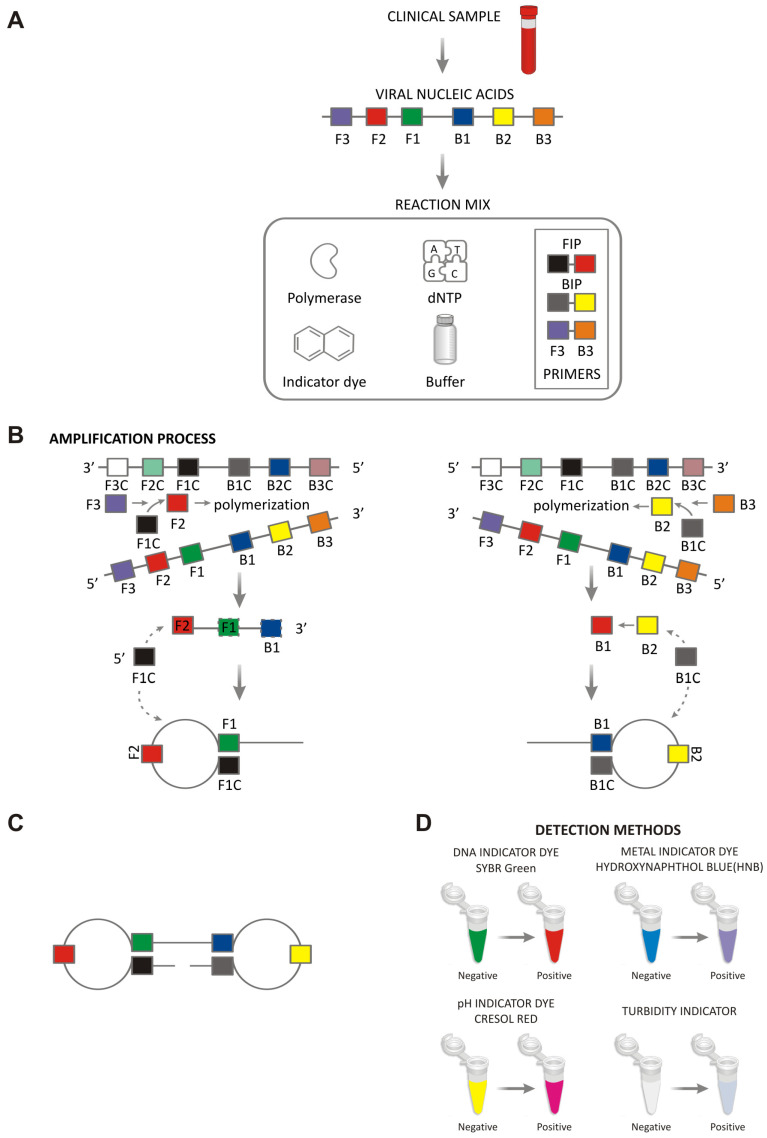
Mechanism of loop-mediated amplification (LAMP). (**A**) Clinical sample collection and extraction of viral nucleic acids. (**B**) Preparation of the LAMP master mix containing primers (FIP, BIP, F3 and B3), DNA polymerase, dNTPs, buffer, and an indicator dye, followed by isothermal amplification. (**C**) Strand displacement synthesis and self-priming amplification generate characteristic looped DNA structures and continuous amplicon production. (**D**) Amplification readout using visual or fluorescence-based detection methods, including DNA-intercalating dyes (e.g., SYBR Green), metal indicator dyes (e.g., hydroxynaphtol blue), pH-sensitive dyes (e.g., cresol red), or turbidity measurement.

The LAMP mechanism consists of two phases: a non-cycling and a cycling phase. During the non-cycling phase (initial strand synthesis), the FIP binds to the target DNA at its F2 portion and the BST polymerase initiates DNA synthesis. The enzyme starts DNA polymerization by the FIP site creating a complementary strand. The BST polymerase lacks 5′-3′ exonuclease activity, which is crucial for strand displacement, ensuring that newly synthesized strands are not degraded. The F3 primer binds to the F3 region of the target DNA and starts DNA synthesis, displacing the DNA strand created by the FIP. The displaced strand forms a loop at its end due to the presence of an F1c sequence fragment and becomes a target for further amplification. At that time, the BIP binds to the B2 region of the newly created strand and starts DNA synthesis. The outer B3 primer binds to the target and initiates strand displacement, forming another loop at the opposite site. At this conformation, the newly synthesized DNA has a dumbbell-shape structure that is closed at both ends. In the cycling phase, the looped DNA serves as a template for continuous self-primed amplification. Therefore, the looped ends provide free 3′-ends for the BST polymerase to start replication without the need of any primers and all displaced strands become templates for further amplification. In this stage, the reaction acquires exponential character as both the template and the displaced strands are amplified simultaneously, with multiple starting points for polymerase activity. In a short time, LAMP reaction produces large quantities of target DNA in the form of concatemers and complex loop structures. The mechanism of LAMP is presented in Figure 1 [26,27,28].

Several approaches are available for detecting positive LAMP reactions, all of which rely on physicochemical changes associated with extensive DNA amplification. These methods include turbidity measurement, colorimetric detection using metal-ion indicators or pH-sensitive dyes, fluorescence-based detection using DNA-binding dyes, and post-amplification analysis by gel electrophoresis [8,29]. Turbidity-based detection results from the accumulation of insoluble magnesium pyrophosphate, a by-product of deoxynucleotide incorporation during DNA synthesis, which increases optical density in positive reactions [30]. Although effective, this approach typically requires dedicated instrumentation and is therefore less suitable for point-of-care or field applications. Colorimetric detection methods enable direct visual interpretation and are particularly advantageous in resource-limited settings. Metal-ion indicators, such as hydroxynaphthol blue, report LAMP through changes in free magnesium ion concentration, typically shifting color from blue to violet in positive reactions [8]. pH-sensitive dyes (e.g., phenol red, bromothymol blue, cresol red) exploit the proton release associated with nucleotide incorporation, leading to a decrease in pH and a corresponding color change as amplification proceeds [31]. Fluorescent detection relies on DNA-intercalating or DNA-binding dyes, including SYBR Green I and related compounds, which emit fluorescence upon binding double-stranded DNA [32]. Although agarose gel electrophoresis allows confirmation of LAMP products through characteristic ladder-like banding patterns, it entails a tube opening and the handling of large quantities of amplified DNA, substantially increasing the risk of carryover contamination and reaction time increase [27].

## 3. LAMP Compared to Real-Time PCR

One of the most important advantages of LAMP compared to qPCR is its cost-effectiveness and simplicity, stemming from its minimal equipment and reagent requirements. In contrast, real-time PCR (qPCR) depends on advanced thermal cyclers with several optical channels that offer precise temperature control during the cycling and fluorescence detection [27]. Time efficiency further differentiates LAMP from qPCR. LAMP reactions typically yield results within 15–30 min, whereas qPCR processes often require ~2 h to complete. This rapid turnaround time is particularly beneficial in health emergencies, such as outbreaks, where timely diagnosis plays a crucial role in controlling the disease spread [33]. Both LAMP and qPCR can demonstrate high analytical sensitivity and specificity, enabling the detection of low levels of target nucleic acids under optimized conditions [32].

Rapid visualization of the amplified products represents another advantage of LAMP. Unlike qPCR, which relies on optical systems and fluorescence detection to determine positivity, LAMP enables direct visualization of amplification through colorimetric changes. This feature simplifies interpretation and reduces the need for specialized technical expertise, broadening its applicability in decentralized settings [26]. Moreover, LAMP design is particularly advantageous for point-of-care testing and field applications, as it requires minimal infrastructure, enabling successful application in remote and resource-constrained regions [34].

Despite these advantages, LAMP presents several important limitations. Primer design is substantially more complex than in qPCR, as LAMP requires multiple primers recognizing six to eight distinct regions of the target sequence, considerably increasing assay design complexity and optimization demands. The use of long primer sequences also elevates the risk of primer–primer interactions, non-specific amplification, and false-positive results, particularly when targeting genetically variable pathogens. This is especially important in settings involving the detection of emerging pathogens or genetic variants, where limited genomic characterization may hinder rapid primer design and increase the risk of non-specific amplification. LAMP is inherently more prone to non-specific signal generation because amplification relies solely on primer annealing and strand displacement, whereas qPCR hydrolysis probes provide an additional layer of specificity.

Another challenge is an accurate absolute quantification of viral load using LAMP due to the non-linear nature of the reaction and the early onset of amplification plateau. In contrast to qPCR, isothermal amplification lacks a well-defined exponential phase that can be modeled across a broad dynamic range. Nevertheless, recent methodological advances have enabled semi-quantitative interpretation through real-time monitoring strategies, including fluorescence- and turbidity-based detection coupled with analysis of time-to-threshold (Tt) values. In this framework, the time required for reaction turbidity to exceed a predefined threshold is inversely related to the initial template concentration. This adaptation permits quantitative assessment of low-abundance targets with high precision across a relatively wide input range [35]. Although this approach supports only approximate estimation rather than absolute quantification, such resolution is sufficient for patient triage, outbreak surveillance, and decision-making in field or emergency settings.

A prominent problem of LAMP is the occurrence of false-positive-reactions. False-positives in LAMP can arise from two different classes of factors: intrinsic methodological properties of the reaction and extrinsic workflow-related causes. Intrinsically, LAMP is characterized by exceptionally high amplification efficiency and the use of multiple primers, features that increase susceptibility to non-specific amplification and primer–primer interactions, particularly during the later stages of the reaction. These structural characteristics are inherent to the LAMP methodology and impose fundamental constraints on reaction specificity, especially in contexts where assay design expertise or molecular optimization capacity is limited [6,36]. In contrast, carryover contamination is predominantly a workflow-related issue arising from the release and subsequent re-amplification of LAMP products within the laboratory environment, particularly when assays involve open-tube handling or post-amplification processing. This form of contamination can be effectively mitigated through the use of closed-tube reaction formats, strict physical separation of pre- and post-amplification workspaces, rigorous decontamination procedures, and the incorporation of dUTP/uracil-DNA glycosylase (UDG) treatment [36,37,38].

In contrast to qPCR, which enables robust multiplex detection through differentially labeled fluorescent probes, multiplex LAMP assays are technically challenging due to primer complexity and increased risk of cross-reactivity. Therefore, the most successfully validated multiplex LAMP assays remain restricted to duplex or triplex formats [36], but they also require extensive empirical optimization of primer concentrations and reaction conditions. Although emerging strategies—including spatial or digital partitioning and probe-based amplicon detection—are gaining traction as potential solutions [39], LAMP multiplexing remains substantially more complex and less scalable than singleplex implementations. Consequently, caution is warranted in the near-term deployment of highly multiplexed LAMP assays [40].

qPCR remains a cornerstone for high-throughput diagnostics in research and laboratory settings, while LAMP offers a practical and scalable complementary alternative for public health emergencies in low-resource environments. Its affordability, speed, and minimal equipment requirements can offer large-scale screening during outbreaks, caused by pathogens such as dengue or influenza, particularly where conventional molecular infrastructure is limited.

## 4. LAMP for Diagnosing Epidemic and Pandemic Viral Threats in Resource-Limited Settings: Dengue and Influenza

### 4.1. LAMP for Detection of Influenza Viruses

Influenza virus provides a suitable model for assessing LAMP performance, owing to its distinct genomic architecture and evolutionary dynamics. Influenza viruses possess a segmented, negative-sense RNA genome and exhibit exceptionally high genetic variability driven by antigenic drift and genomic reassortment [21]. Highly sensitive influenza diagnostics depend critically on continuous surveillance of circulating lineages, as primer–target mismatches can rapidly emerge following the appearance of novel strains. These features pose substantial challenges for molecular diagnostic approaches, including LAMP. Moreover, influenza imposes a considerable global public health burden through recurrent seasonal epidemics and episodic pandemics, underscoring the need for rapid, adaptable, and scalable diagnostic tools [22]. The evaluation of LAMP in the context of influenza therefore illustrates its applicability to rapidly evolving RNA viruses under dynamic outbreak and surveillance conditions [41].

LAMP has been extensively validated for influenza A viruses of veterinary importance, where rapid, affordable and deployable diagnostics are essential. Its principal application has been in the detection of avian influenza viruses in poultry populations, supporting on-site outbreak investigation and herd-level management. LAMP assays targeting highly pathogenic H5 clade 2.3.4.4.b enable detection within 30 min with high analytical sensitivity [42]. For H5N1, LAMP demonstrates a tenfold higher sensitivity than conventional RT–PCR, achieving a limit of detection of 0.1 PFU compared to 1 PFU for RT–PCR [43]. Point-of-care LAMP platforms for avian H5 and H9 subtypes further demonstrate analytical sensitivities of 100 copies per reaction for H5 and 1000 copies per reaction for H9 within 45 min [44]. In swine, LAMP assays have been optimized for the detection of H3 influenza viruses, achieving detection of 1 PFU within 45 min, compared with 10 PFU by RT–PCR [45]. Swine are a key reservoir for viral reassortment and the emergence of pandemic variants. Through targeting conserved regions such as the matrix gene [46,47], LAMP enables rapid processing with high specificity, making it suitable for herd-level screening and large-scale veterinary surveillance initiatives [47]. Collectively, these data demonstrate that LAMP constitutes a sensitive, rapid and cost-effective molecular diagnostic tool for veterinary field applications, particularly in livestock and poultry settings. Its robustness, minimal equipment requirements and suitability for deployment outside centralized laboratories underscore its value for veterinary outbreak response and routine field surveillance [48].

LAMP has also demonstrated strong clinical utility for the diagnosis of human influenza viruses with pandemic potential, particularly in point-of-care settings [21]. Seasonal and pandemic influenza A subtypes, including H1N1 and H3N2, as well as influenza B, can be detected within 12–25 min with high analytical sensitivity (as low as 1–50 copies per reaction) and reported specificities approaching 100% [49,50] Multiplex LAMP assays further enable simultaneous detection of influenza A and B with limits of detection around 10^2^ copies/µL [51]. Importantly, clinical LAMP platforms have been developed for the concurrent identification of multiple respiratory pathogens in a single reaction, including influenza A/B in combination with adenovirus, parainfluenza viruses, rhinovirus, respiratory syncytial virus and SARS-CoV-2 [52,53,54], underscoring their applicability in routine clinical respiratory diagnostics.

The 2009 pandemic H1N1 virus exemplifies the clinical relevance of LAMP-based detection [55]. Assays targeting the matrix gene achieve sensitivities of 20–60 copies per reaction within 20–60 min, with reported clinical sensitivities of 96.3–100% and specificities of 88.9–98.25%, while discriminating H1N1 from H3N2 and other respiratory viruses [56,57,58]. Notably, simplified “pocket” LAMP formats using portable heat sources have been proposed for rapid bedside or outpatient diagnosis [59].

Building on these clinical applications, LAMP has also been developed for the detection of influenza A viruses with heightened pandemic risk, including H7N9. Human infections with H7N9 have been associated with substantial mortality in specific demographic groups [31], underscoring the need for rapid and reliable clinical diagnostics. H7N9-specific LAMP assays achieve detection limits of 5–10 copies/reaction within 23–30 min, without cross-reactivity to other pandemic influenza A subtypes such as H1N1, H3N2 or H5N1, demonstrating high analytical specificity [60,61,62]. Reported sensitivity was higher compared to conventional RT–PCR (5 × 10 copies/reaction vs. 5 × 10^2^ copies/reaction, respectively) [61].

Together with the previously described assays for H1N1 and H3N2, these data reinforce that LAMP is a rapid, sensitive and clinically robust molecular diagnostic platform for frontline detection, differential diagnosis and epidemiological surveillance of influenza A viruses with pandemic potential [62].

### 4.2. LAMP for the Detection of Dengue Serotypes

Dengue fever is caused by the dengue viruses (DENV serotypes 1–4) and currently, from a public health perspective, is the most important arbovirus infection in the world, disproportionately affecting low- and middle-income countries, where limited access to reliable diagnostics hinders early case detection and timely outbreak response, contributing to elevated morbidity and mortality. The symptoms may vary, from an asymptomatic or mild infection to severe hemorrhagic disease related to high lethality [63]. During 2024, there was reported the largest DENV outbreak in Brazil with more than 6 million probable cases and more than 4000 DENV associated deaths [64]. In this context, rapid, affordable, and equipment-independent diagnostic approaches are critically needed, positioning LAMP as a promising tool for decentralization surveillance and real-world outbreak management [65].

DENV constitutes a particularly stringent and informative model for the evaluation of LAMP-based diagnostics. As a positive-sense RNA virus with a high mutation rate, DENV places substantial constraints on primer design and poses significant challenges for achieving robust assay specificity and sensitivity [66,67]. These challenges are further exacerbated by the co-circulation of multiple serotypes, major lineages, and genotypes in hyperendemic regions, which can markedly compromise the performance of molecular diagnostic assays.

The detection of DENV by LAMP has shown high sensitivity and specificity. The LAMP for DENV-1–4 showed 93–100% specificity and 86.3–100% sensitivity, and the limit of detection was low, with a detection of 3.5–4 copies/reaction for 25 min [68,69,70,71]. Lyophilized forms of DENV LAMP have also been proposed and they are highly suited for field work. These tests have also shown a high sensitivity and specificity of 100% and 92%, respectively, compared to ELISA techniques with a short turnaround time [72]. Due to the genetic divergence between the DENV serotypes, a mismatch-tolerant LAMP has been developed. The enzyme used in this reaction, high-fidelity DNA polymerase, removed the mismatched bases at the 3′-end of the primers, resulting in excellent tolerance to mutations. This reaction also showed an excellent limit of DENV detection, with a sensitivity of 74, 252, 78 and 35 RNA copies/reaction for DENV-1, DENV-2, DENV-3 and DENV-4 [73].

LAMP has also been applied for multiplexed detection of arboviruses in mosquitos, which can be a suitable tool for monitoring transmitting vectors and the risk of outbreaks. The detection of LAMP DENV and West Nile virus RNA was possible for 45 min in mosquito saliva, without the need of nucleic acid extraction [74]. The combination of LAMP for DENV, Zika and Chikungunya in the form of LAMP can be applied as a rapid diagnosis during outbreaks for the correct etiological diagnosis, due to the similarity of the clinical symptoms of these infections [75]. For that reason, LAMP for DENV detection can revolutionize the detection of this arbovirus in resource-limited countries, point-of-care facilities, during field work in remote areas and areas with a high endemicity of this virus [76].

## 5. Integration of LAMP in Limited-Resource Countries

**Figure 2:**
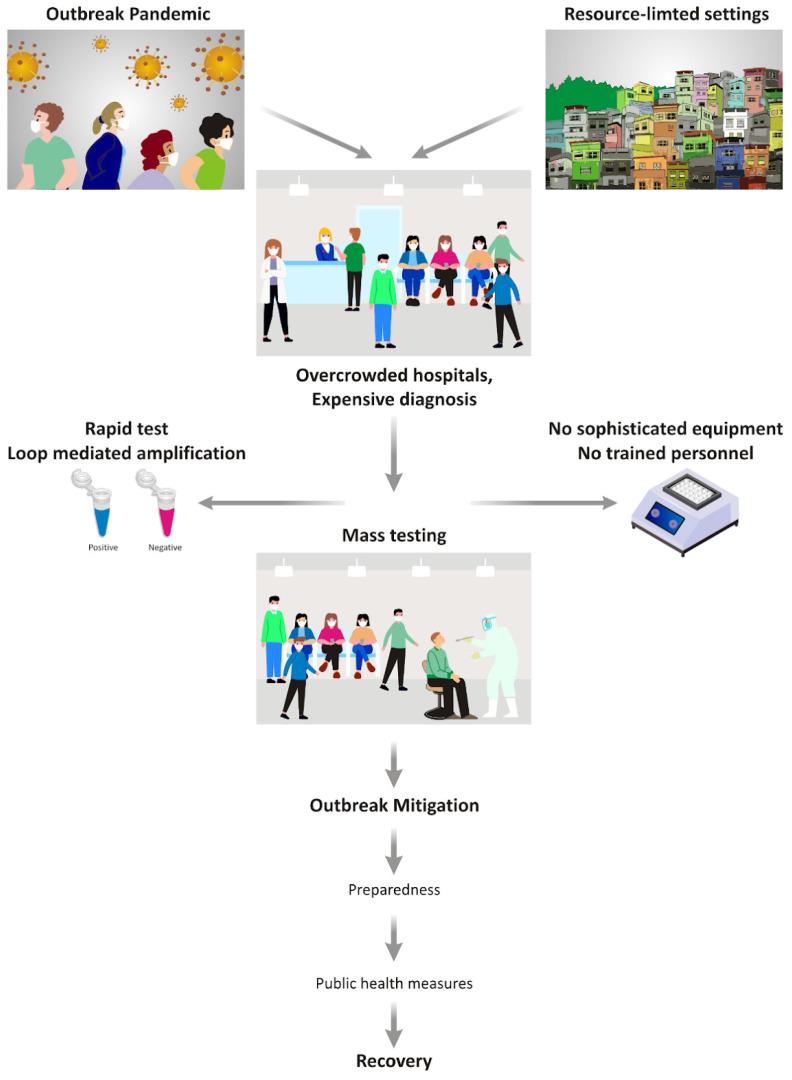
Outbreak impact and potential application of LAMP in resource-limited settings. Large epidemics and pandemics can overwhelm healthcare systems in resource-limited countries, leading to overcrowded hospitals and limited diagnostic capacity. Rapid, point-of-care tests that do not require complex equipment or highly trained personnel are therefore essential. An optimized LAMP assay, requiring only sample addition and simple result interpretation, could enable large-scale testing. Widespread diagnosis would facilitate timely isolation, implementation of non-pharmaceutical interventions, and appropriate treatment, thereby reducing outbreak impact and improving preparedness for future events.

Limited healthcare infrastructure, including diagnostic facilities, is one of the most significant factors predisposing resource-limited countries to increased vulnerability to infectious diseases with epidemic potential. It is now evident that viruses with pandemic and epidemic potential exert a greater impact in resource-constrained environments. Influenza results in higher mortality rates in resource-limited countries, primarily due to decreased access to healthcare, inadequate healthcare infrastructure, limited diagnostic capabilities, and poor availability of healthcare services. Furthermore, higher prevalence rates of HIV, tuberculosis, malnutrition, and lack of vaccinations significantly contribute to the severe impact of influenza in these settings [77]. Therefore, enhanced preparedness can mitigate the effects of outbreaks in such contexts. Unlike in developed countries, pharmaceutical interventions and vaccines are of limited use in resource-limited countries [78]. One of the most pressing issues is limited access to diagnostic tests due to insufficient laboratory infrastructure. Therefore, the development of cost-effective, simple, and rapid diagnostic tests is essential to mitigate outbreaks and support decision-making during public health emergencies. In this context, LAMP has emerged as one of the most promising tools for the specific diagnosis of infections in resource-limited settings (Figure 2).

The implementation of LAMP in resource-limited settings is primarily driven by the intrinsic characteristics of the assay-simplicity and cost-effectiveness. Furthermore, many of the necessary reagents can be locally produced, thereby further reducing costs and minimizing dependence on centralized biotechnological suppliers [79,80,81].

The rapid speed of LAMP is particularly advantageous for clinical decision-making in low-resource contexts, facilitating timely point-of-care diagnostics [32,80]. Moreover, the assay exhibits reduced susceptibility to inhibitors commonly found in clinical samples, making it especially relevant in settings where extensive sample processing, and especially extraction of nucleic acids, is not feasible. LAMP can be performed directly on crude biological specimens—such as saliva, urine, or blood—without requiring nucleic acid extraction [7,80].

Another key advantage of LAMP is its adaptability to field conditions. Its platforms can be miniaturized and battery-operated. The reaction permits colorimetric detection, obviating the need for fluorescent probes and optical detection [82]. The high sensitivity and specificity of LAMP further support its use in low-resource settings, especially for the detection of high-burden viruses [83]. Multiplexing strategies have also been successfully integrated into LAMP platforms, adding value for syndromic surveillance during outbreaks of arboviral infections, which often present with overlapping clinical symptoms [84]. These features make LAMP exceptionally well-suited for rapid outbreak response and decentralized surveillance, particularly for high-burden pathogens such as arboviruses or respiratory viruses. The application of LAMP enables timely implementation of containment measures and therapeutic interventions where available. In Table 1, we present the characteristics of LAMP compared to other isothermal reactions and in particular for application as a diagnostic tool in resource-limited settings:

Despite the inherent advantages of LAMP for use in resource-limited settings, several challenges remain. Core components of the reaction mixture, including BST polymerase and certain master mix formulations, typically require cold-chain storage to maintain enzymatic stability. In low-resource settings, this may introduce additional logistical complexity and cost. Accordingly, the integration of entirely new diagnostic technologies into national healthcare systems may encounter regulatory obstacles and bureaucratic delays, often resulting in protracted approval timelines that hinder timely adoption within existing diagnostic and procurement frameworks. This is one of the main reasons for the limited number of diagnostic trials implementing LAMP as a routine detection tool in settings with restrained resources. At present, phase III clinical trial data for LAMP-based diagnostics in limited-resource countries remain limited, and the trials summarized in Table 2 represent the entirety of publicly available late-stage clinical evaluations of LAMP assays irrespective of pathogen class.

For that reason, from an implementation perspective, the deployment of LAMP in resource-constrained settings typically follows a staged pathway. Initial integration often relies on commercially available or pre-validated kit formats, including lyophilized or stabilized reagent mixes that reduce cold-chain dependency and simplify handling. Storage strategies may range from refrigerated distribution at regional hubs to ambient-temperature transport of lyophilized kits with desiccant-protected packaging for peripheral facilities. At the point of use, minimal equipment configurations—such as portable heat blocks or battery-powered incubators—enable decentralized testing. Over time, implementation may transition toward locally assembled or in-house assays where supply chains permit, provided that standardized validation and quality assurance measures are in place. This phased approach balances operational feasibility with sustainability. Collectively, these innovations have significantly improved the application and practicality of LAMP-based diagnostics [7,15,93].

Furthermore, although LAMP is operationally simpler than conventional and real-time PCR techniques, its effective use still demands knowledge in molecular biology. This necessitates targeted training of laboratory personnel and broader capacity-building initiatives to ensure accurate implementation and interpretation of results. Additional technical barriers include protocol variability across laboratories, the frequent use of in-house assay designs lacking standardized regulatory benchmarks, and a heightened risk of contamination due to open-tube procedures.

Further perspectives for the implementation of LAMP in resource-limited countries include the lyophilization of reagents and the development of formulations that do not require a cold chain. Lyophilized LAMP reagents offer practical advantages for field deployment, including improved thermal stability during storage and transport, reduced dependence on continuous cold-chain logistics, and simplified assay setup [94]. However, stability remains influenced by humidity exposure and packaging integrity, and long-term storage in high-temperature or high-moisture environments may still require protective desiccation and barrier materials [95]. Thus, while lyophilization enhances operational feasibility, it does not fully eliminate environmental constraints. These advances make LAMP more suitable for field use and have the potential to transform diagnostic algorithms in settings with limited infrastructure.

Another promising direction is the integration of LAMP with emerging technologies such as CRISPR/Cas systems, enabling the design of highly specific and sensitive diagnostic assays that might overcome common issues with non-specific LAMP reactions and false-positive colorimetric changes [96]. However, CRISPR–LAMP assays should be viewed as an enhancement of conventional LAMP rather than as a direct diagnostic alternative. In these integrated workflows, LAMP enables rapid and sensitive target amplification, while CRISPR-based detection introduces an additional layer of sequence-specific recognition [97] that can mitigate false-positive signals arising from non-specific amplification [98]. This added specificity is particularly advantageous in applications requiring discrimination among closely related targets, where LAMP primer design alone may be insufficient [99]. However, the incorporation of CRISPR detection also increases assay complexity, reagent burden, and overall cost, potentially limiting feasibility in low-resource or point-of-care settings [38,100]. In such contexts, well-optimized singleplex LAMP assays employing closed-tube detection formats may remain the more practical option, whereas CRISPR–LAMP approaches are better suited for high-stakes diagnostic applications demanding maximal specificity.

Recent advances in computational primer design are begging to address some of the specific limitations of LAMP, especially related to primer design. Machine learning-based models and in silico simulation pipelines have been proposed to predict primer performance, secondary primer structures, primer–primer interaction risks and the specificity of genomic binding. These methods may reduce the operational burden associated with LAMP optimization and consequent field or diagnostic employment. Such computational frameworks are particularly relevant in outbreak settings involving emerging viruses or rapidly evolving pathogens, where a rapid incorporation of a diagnostic test is essential. The incorporation of predictive bioinformatic approaches into LAMP assay design pipelines may enhance reliability, shorten development turnaround times and support more agile responses in resource-limited settings [101,102,103,104].

Additionally, LAMP shows the possibility to be adapted for use by non-specialist personnel through simplified, user-friendly formats, including colorimetric detection and smartphone-based readouts. This democratizes access to molecular diagnostics, allowing for its application even in community-based settings without the need for trained laboratory staff. Due to its rapid turnaround time, LAMP can significantly reduce diagnostic delays, thereby contributing to more effective outbreak containment, with substantial epidemiological and economic benefits. This growing utility is reflected in the inclusion of LAMP in WHO policies, such as for the diagnosis of pulmonary tuberculosis, further highlighting the shift toward regulatory acceptance and the incorporation of LAMP into national diagnostic algorithms (https://www.who.int/publications/i/item/9789241511186 accessed on 21 May 2025).

Delayed or inadequate diagnosis of infectious diseases remains a major barrier to effective surveillance and outbreak control, particularly during epidemics and in resource-limited settings. In this context, LAMP is well-positioned to address near-term diagnostic needs by enabling rapid, sensitive, and equipment-light detection of pathogens at or near the point of care. Over the next 5–10 years, realistic deployment scenarios for LAMP include decentralized testing in primary healthcare facilities, field-based surveillance of arboviral, respiratory and neglected tropical diseases and rapid screening during emergency outbreak response where time-to-result is critical. In these contexts, the primary value of LAMP lies in qualitative presence–absence testing for rapid screening and early outbreak detection. At the same time, it is important to recognize the limitations of this method: LAMP is not suited for applications requiring high-precision quantitative measurements, broad and complex multiplexing, or fine-scale discrimination among numerous closely related targets. Acknowledging both its strengths and limitations, LAMP should be viewed not as a universal replacement for established molecular platforms, but as an affordable diagnostic tool for rapid detection, surveillance, and triage under constrained conditions.

## Figures and Tables

**Table 1:** Comparison between LAMP and other isothermal amplification methods.

	Loop-Mediated Isothermal Amplification (LAMP) [7]	Recombinase Polymerase Amplification (RPA) [85]	Nucleic Acid Sequence-Based Amplification (NASBA) [86]	Helicase-Dependent Amplification (HDA) [87]
Reaction Time	30–60 min	10–30 min	60–90 min	60 min
Optimal Reaction Temperature	60–65 °C	37–42 °C	40–50 °C	65 °C
Amplification Enzymes	BST polymerase	Recombinase and DNA polymerase	AMV Reverse transcriptase, RNAse H, t7 RNA polymerase	Single-stranded binding protein (SSB), helicase and DNA polymerase
Number of Primers	4 to 6 primers	2 primers	2 primers	2 primers
Equipment	Heat block or water bath	Heat block	Heat block	Heat block
Field Application	Good ability	Good ability	Bad ability	Good ability [87]
Revelation of Positive Results	Colorimetric, fluorometry or turbidity	Fluorescence and lateral flow	Molecular beacon [88]	Fluorescent DNA binders [89]

**Table 2:** All publicly available phase III clinical trials for LAMP-based diagnosis in low- and middle-income countries.

Study	Disease	Countries	Phase	Target Population
DIAGMAL [90]	Malaria	Ethiopia, Sudan, Kenya, Namibia	Phase III	Adults and children in endemic areas
LAMP4Yaws [91]	Yaws	Cameroon, Ghana, Ivory Coast	Phase III	School-aged children and rural communities
TB-LAMP (WHO) [92]	Tuberculosis	Uganda, Tanzania, Bangladesh	Phase III	Symptomatic patients with suspected TB

## Data Availability

No new data were created or analyzed in this study. Data sharing is not applicable to this article.
